# In Silico Methods for the Discovery of Kv7.2/7.3 Channels Modulators: A Comprehensive Review

**DOI:** 10.3390/molecules29133234

**Published:** 2024-07-08

**Authors:** Claudio Stagno, Francesca Mancuso, Tania Ciaglia, Carmine Ostacolo, Anna Piperno, Nunzio Iraci, Nicola Micale

**Affiliations:** 1 Department of Chemical, Biological, Pharmaceutical and Environmental Sciences (CHIBIOFARAM), University of Messina, Viale F. Stagno d‘Alcontres 31, 98166 Messina, Italyfrancesca.mancuso@unime.it (F.M.); anna.piperno@unime.it (A.P.); 2 Department of Pharmacy, University of Salerno, Via G. Paolo II, 84084 Fisciano, Italy; tciaglia@unisa.it (T.C.); costacolo@unisa.it (C.O.)

**Keywords:** potassium channel, Kv7, KCNQ, molecular docking, molecular dynamics, antiepileptic, epileptic encephalopathy

## Abstract

The growing interest in Kv7.2/7.3 agonists originates from the involvement of these channels in several brain hyperexcitability disorders. In particular, Kv7.2/7.3 mutants have been clearly associated with epileptic encephalopathies (DEEs) as well as with a spectrum of focal epilepsy disorders, often associated with developmental plateauing or regression. Nevertheless, there is a lack of available therapeutic options, considering that retigabine, the only molecule used in clinic as a broad-spectrum Kv7 agonist, has been withdrawn from the market in late 2016. This is why several efforts have been made both by both academia and industry in the search for suitable chemotypes acting as Kv7.2/7.3 agonists. In this context, in silico methods have played a major role, since the precise structures of different Kv7 homotetramers have been only recently disclosed. In the present review, the computational methods used for the design of Kv.7.2/7.3 small molecule agonists and the underlying medicinal chemistry are discussed in the context of their biological and structure-function properties.

## 1. Introduction

In the last years, the Kv7 (known also as KCNQ) potassium ion channels have become an object of great interest, due to their association with several rare diseases such as developmental and epileptic encephalopathies (DEE), benign familial neonatal seizures (BFNSs), neonatal epileptic encephalopathy (NEE), mental retardation, long-QT syndrome, short-QT syndrome, atrial fibrillation, and diabetes mellitus [1,2,3,4,5,6,7]. Many biological processes of excitable cells, such as regulation of apoptosis processes, cell growth and differentiation, release of neurotransmitters and hormones, maintenance of cardiac activity, etc., depend on cellular repolarization upon efflux of cations, among which K^+^ is the predominant one. Indeed, the potassium channels are widely expressed in human organisms, and they are structurally diversified considering their function and distribution. The main classes of known K^+^ channels are the Ca^2+^-activated channels (KCa), the inwardly rectifying channels (Kir), the two-pore domain (K2P), and the voltage-gated (Kv) channels [8].

Kv channels form the most abundant and diversified group, composed of 12 distinct families, namely, Kv1–Kv12. Kv7 are singular among K^+^ channels, since four of the five existing subtypes (Kv7.1–Kv7.5, encoded, respectively, by the *KCNQ1*–*KCNQ5* genes) have a widely documented role in the onset of human diseases. The five members of the Kv7 family share many common biophysical characteristics but, despite this, they play different roles in the organism dependent on the subunits they are composed of and on the cellular environment they are found in. Since they were first discovered in cardiac myocytes and neurons, Kv7 channels are often distinct as “cardiac” Kv7 channels (Kv7.1) and “neuronal” ones (Kv7.2–7.5). However, nowadays Kv7 channels are recognized to have a greater physiological impact in many other cell types, such as epithelial and smooth muscle cells [9].

**Figure 1:**
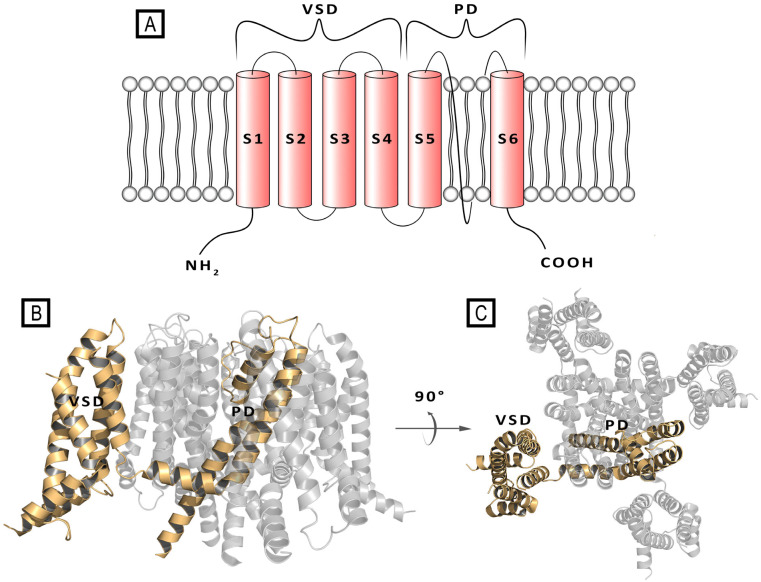
(**A**) Schematic representation of single transmembrane subunit of a Kv7 channel. (**B**) Front- and (**C**) top-view of a crystal structure of the Kv7.2 channel (PDB ID: 7CR0). In both (**B**) and (**C**) panels, a single subunit is represented as wheat cartoon [11].

At the structural level, the Kv voltage-channels assemble as tetramers of identical or different monomers, thus forming homo- or heterotetramers. Each subunit consists of six transmembrane (TM) alpha helix segments (S1–S6) and intracellular N- and C-termini. The TM portion comprised between S1 and S4 helices forms the voltage-sensing domain (VSD), where the S4 segment is crucial for channel gating. The S5 and S6 helices and the interconnecting loop (P-loop) participate in the formation of the pore domain (PD), acting as the ion-conducting pathway, and including a selectivity filter and a water-filled cavity (Figure 1) [9,10,11].

The Kv7 C-termini is longer than the other Kv isoforms and it is organized in four distinct helices (A–D) containing fundamental sites for protein tetramerization and for interaction with determinant endogenous regulators, such as phosphatidylinositol 4,5-bisphosphate (PIP2), syntaxin, calmodulin (CaM), protein kinase C, A-kinase-anchoring proteins, and ankyrin-G [12]. 

Neuronal Kv7 channels produce a low voltage-activated K^+^ current termed as the “M-current”, due to the observation that muscarine inhibited this voltage-sensitive potassium current [13]. M-current suppression causes a drastic increase in neuronal firing, that significantly impacts neuronal functions. Despite its clear physiological effects on neuronal excitability, understanding the molecular mechanism underlying M-current suppression has taken many years and represents a topic of continuous debate. In fact, all proteins belonging to the big Kv family share a similar mechanism of activation but, despite numerous studies conducted, there is still no single model recognized by the scientific community [9]. A detailed knowledge of the atomic structure of Kv channels in the various functional states could clarify the conformational changes that occur in the Kv channel during gating and how these changes are connected to channel opening, closing, and inactivation.

However, the aforementioned represents a major limitation since the transition states between the two final channel conformations (open and closed) are extremely unstable and short-lived, hindering the experimental solving of such structures. Therefore, the numerous models of Kv channel activation described so far are realized on the basis of structural information gained by different experimental approaches (e.g., voltage-clamp fluorometry, mutational analysis, biophysical data) and by the use of in silico techniques [14,15,16,17].

In Kv7 state transitions, the physiological role of co-factor lipid PIP2 emerges: the activation of the G-protein-coupled muscarinic acetylcholine receptors (mAChRs) mediates the activation of phospholipase C, the enzyme responsible for the hydrolysis and depletion of PIP2, that finally results in the inhibition of the neuronal Kv7.2/7.3 channel activity. It is now clear that PIP2 stabilizes the open conformation of the channel, thus increasing the probability of opening. Additionally, in Kv7.1, PIP2 enhances the coupling between the VSD and the PD, making channel opening more efficient in response to action potential changes. Indeed, it was demonstrated by cryo-electron microscopy (Cryo-EM) studies that the binding of PIP2 alters the conformation of the channel with the consequent opening of the pore, changing the helix-loop-helix structure of the S6 subunit and the HA helix, forming a single homogeneous helix [18]. Kv7 channels are also inhibited by other G-protein-coupled receptors including those of bradykinin, substance P, opioids, serotonin, angiotensin, hormone-releasing and luteinizing hormones, as well as by metabotropic glutamate receptors [19]. The transduction ability of Kv7 is also modulated by the ubiquitous Ca^2+^-transducer CaM, which binds to C-termini to drive the tetramerization process and the correct protein folding. Considering the key role of the endogenous small molecules above discussed in the modulation of Kv7 activity, it is a foregone conclusion to wonder whether it is really a simple voltage-dependent channel.

## 2. Chemical and Physio-Pathological Implications of KCNQ Gene Mutations

It is now known that mutations in Kv genes (KCNQ1–5) are related to several disorders like long-QT syndromes (KCNQ1) and a range of neuropsychiatric disorders (KCNQ2–5), ranging from some types of neonatal epilepsy (NEE) and epileptic encephalopathies (DEEs) up to developmental disorders [20,21,22]. Individuals affected by KCNQ-related epileptic and developmental encephalopathy (KCNQ-E), despite the use of antiepileptic drugs (AED) that can affect seizure control, still experience severe developmental delays. As a result, there is an urgent need to develop new therapies that not only address seizures but also developmental problems. It is worth mentioning that most of the Kv7-related epilepsies are related to a Loss-of-Function (LoF) Kv7 phenotype, while other neurological disorders, such as the KCNT1-related ones, are mostly related to Gain-of-Function (GoF) phenotypes. Considering the significant role of the Kv7.2 and Kv7.3 channels in regulating neuronal excitability, channel activation, in the case of LoF phenotypes, represents a promising tool for treating or preventing neurological disorders associated with neuronal hyperexcitability (e.g., epilepsy, neuropathic pain, ischemic stroke, amyotrophic lateral sclerosis). On the other hand, when dealing with GoF-related disorders, channel blockades by inhibitors represents a potential therapeutic opportunity [23,24].

In Kv7.2, the presence of a basic residue in the proximal C-terminus is essential for the activation of the M-channel subunit by PIP2, highlighting the importance of this region in the channel activation mechanism. Mutations affecting the proximal C-terminal region of Kv7.2, especially at residues Arg325 and Lys327, have been shown to disrupt the channel’s response to PIP2, indicating that these specific residues are essential for proper channel functionality. Soldovieri et al. focused their attention on the functional and biochemical consequences exerted by a recurrent variant affecting the Kv7.2 gene (Arg325Gly) that was found in four patients affected by a severe form of neonatal-onset epileptic encephalopathy [25]. The results obtained revealed that the Arg325Gly mutation strongly impaired Kv7.2 channel functionality by reducing channel affinity for the determinant co-factor PIP2. As consequence, this highlights that strategies able to increase the cellular concentration of PIP2 further extend the range of pathogenetic mechanisms exploitable for the identification of personalized therapies for Kv7.2-related epilepsies. 

**Figure 2:**
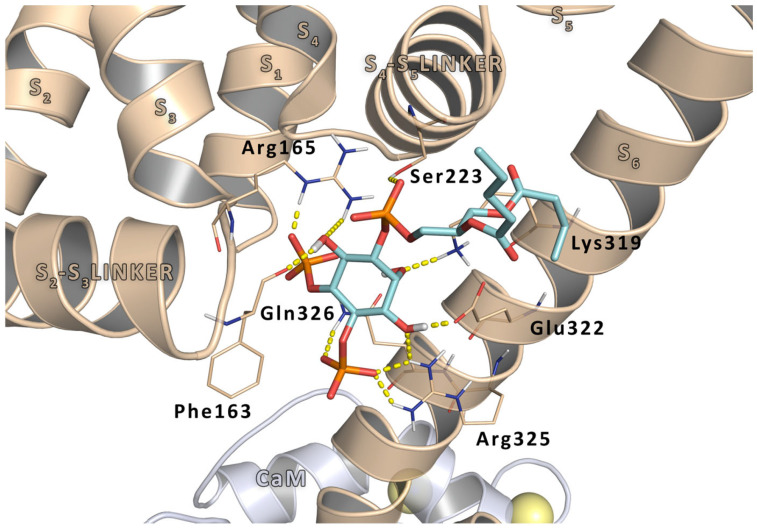
Interaction details of the PIP2 docking pose inside Kv7.2 homology model complexed with calmoduline. The pore portion is represented as wheat cartoon, calmoduline as gray cartoon, and the two Ca^2+^ ions are represented as yellow spheres. Residues involved in ligand interactions are represented as thin sticks. PIP2 is represented as cyan sticks. H-bonds are represented by yellow dashed lines.

The role of Arg325 residue in PIP2 binding has been also investigated by molecular modeling studies. In particular, a Kv7.2 homology model was built based on a Kv7.1 crystal structure of the Kv7.1 proximal C-terminus, including the A and B helices, integrating with the crystal structure [26]. A short-chain derivative of PIP2, dioctanoyl-PIP2 from its co-crystal structure with the Kir 2.2 channel, was then grafted, after alignment, onto this model to define the PIP2 docking space [27]. Thus, molecular docking experiments were performed on this model to find the best scoring for Kv7.2/PIP2 binding conformation. The results revealed that, concerning this region, the negatively charged ligand is involved in a dense network of electrostatic interactions with the side chains of residues located in the S2-S3 linker (Phe163, Arg165), in the S4–S5 linker (Ser223), and in the pre-helix A region (Lys319, Glu322, Arg325, and Gln326) (Figure 2). In particular, the Arg325 side chain interacts simultaneously with the 3′OH and the 4′PO_4_^2−^ of the PIP2. Finally, the stability of the interaction between the phosphorus atom at C4′ of PIP2 and the ζ-carbon of the Kv7.2 Arg325 residue was supported by molecular dynamics experiments, thus providing an explanation, at the molecular level, for the experimentally observed LoF Kv7.2 phenotype.

Ambrosino et al. described the first case of heterozygosity in the Kv7.3 gene in a patient affected by early onset epileptic encephalopathy (EOEE) carrying two Kv7.3 missense mutations (Val359Leu and Asp542Asn) [28]. Various functional experiments highlighted that these Kv7.3 mutations lead to detrimental effects on channel functionality, and that the disease pathogenesis in EOEE patients may correlate with a reduced affinity of the channel for PIP2. These results have been investigated in silico to gain molecular insight into the PIP2/Kv7.3 interaction, using homology modeling, molecular docking, and MD simulations. A homology model of a Kv7.3 homotetramer was built using the crystal structure of the Kv7.1 proximal C-terminus, including the A and B helices [26].

**Figure 3:**
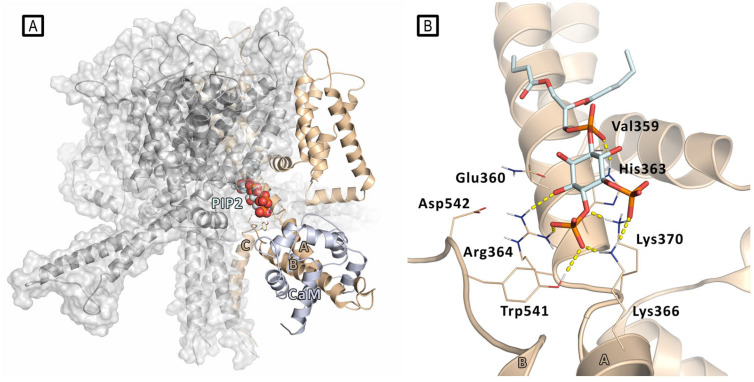
(**A**) Docking-predicted pose of PIP2 (represented in spheres) bound to a model of Kv7.3 tetramer (the single PIP2-bound subunit is represented in wheat cartoon, the other subunits in gray) with the presence of calmodulin (CaM) molecule (represented as light blue cartoon). (**B**) Details of PIP2 docking pose into a Kv7.3 subunit. The residues involved in PIP2/Kv7.3 interactions are represented as thin sticks. H-bonds are represented by yellow dashed lines.

The two pathogenic mutations affect residues located about 200 amino acids apart in the long C-terminus of the Kv7.3 primary structure; indeed, the residue Val359 falls in the linker between A-helix and S6, whereas the Asp542 lies in the portion comprised between the B and C helices. The modeling of the Kv7.3 homotetramer highlighted that these two regions are spatially close and in proximity of the pore bottom (Figure 3); this happens as a consequence of the anti-parallel orientation assumed by the A and B helices. In this model, the protein region at the interface between the C-terminus and the TM domains, where both Val359 and Asp452 are located, is a crucial region for interaction with the negatively charged lipid PIP2 and the modulation of channel function. To identify the best-scoring Kv7.3/PIP2 conformation, a short chain derivative of PIP2 (dioctanoyl-PIP2) was subjected to docking analysis onto this model. The results showed that the PIP2 molecule establishes a tight network of electrostatic interaction involving the side chains of aminoacidic residues located in the pre-helix A region (Gln360, His363, Arg364, Lys366, Lys370) and in the B–C inter-helical region (Tyr541). Its hydrophobic tail faces toward a lipophilic cleft delimited by the non-polar residues Leu355, Ala356, Leu357, and Val359. MD simulations were run in the explicit membrane and solvent to confirm the stability of the found interactions. By this model, Val 359 was thus found to directly interact with PIP2 and mutation of this residue was then thought to directly affect PIP2/Kv7.3 affinity. On the other hand, PIP2/Asp542 interaction is mediated by Arg364, so neutralization of the Asp542 negative charge by mutation to Asn might displace Arg364, with a subsequent weakening of the interaction with PIP2. It is worth noting that Kv7.3 Arg364 corresponds to Kv7.2 Arg325, which was previously reported to affect the affinity of Kv7.2 for PIP2 [25].

PIP2 is not the only endogenous direct activator of Kv channels. In 2018, R. W. Manville et al. reported the first description of a direct activation of Kv7 channels by endogenous neurotransmitters, revealing a novel mechanism to control neuronal excitability [8]. Indeed, Kv7.2 is particularly widespread in GABAergic neurons, especially those involved in the regulation of neuronal network oscillations and synchronization [29]. Kv7.2/7.3 channels in pre-synaptic neurons are suggested to modulate γ-aminobutyric acid (GABA) and glutamate release and are thought to act pre- and post-synaptically to inhibit neuronal excitability. The putative binding mode of GABA on Kv channels was predicted by molecular docking simulation conducted by SwissDock (using CHARMM forcefields). The results of this in silico investigation, supported by experimental studies, suggested that Kv7.3-Trp265, which is a key residue for RTG binding, forms part of a high-affinity binding pocket for GABA and its relative metabolites, whose binding induces channel activation and consequent membrane hyperpolarization.

## 3. In Silico Approaches for Retigabine Binding Site Modeling

**Figure 4:**
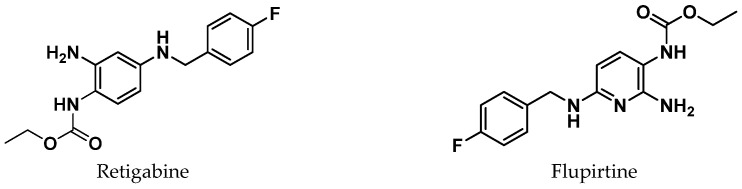

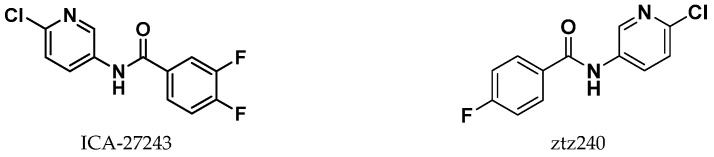
2D-chemical structure of previously reported Kv7.2/7.3 activators [11,30,31].

**Figure 5:**
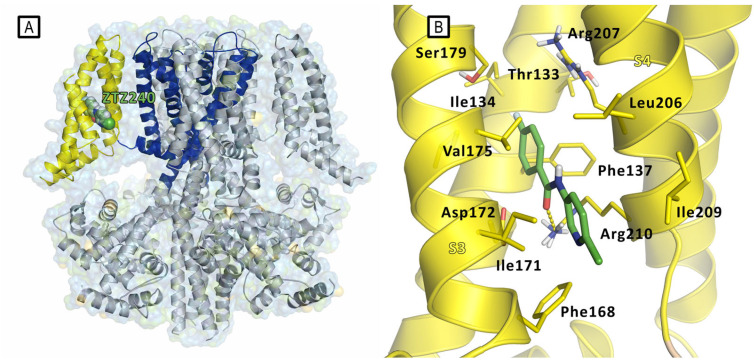
(**A**) Representation of the Cryo-EM structure Kv7.2 in complex with ztz240 (PDB ID: 7CR1). Kv7.2 is represented by cartoons and surface, while ztz240 is represented by green spheres inside the VSD binding site. Cartoons are colored as follows: PD of one subunit is colored in blue, while the VSD of the same subunit is colored in yellow. The other subunits are represented in gray. (**B**) Experimental bound conformation of ztz240 (green sticks). Residues of the pocket are represented by yellow sticks, and include Phe137, Asp172, and two gating charge residues Arg207 and Arg210 in the side pocket comprised between S3 and S4 helices (VSD). Hydrogen bonding (yellow dashed line) is found between the ligand and the side chain of Arg210 [11].

In recent years, several molecular modeling studies have been carried out to explore the differences at the binding sites among all the Kv7 channel subfamilies and to improve knowledge on the molecular mechanism mediated by endogenous and exogeneous activators. Several synthetic compounds that act as Kv7.2 or Kv7.3 activators, preventing abnormal or excessive neuronal firing, have been described so far (Figure 4). Among them, retigabine (RTG, also noted as ezogabine) (ethyl N-[2-amino-4-[(4-fluorophenyl)methylamino]phenyl]carbamate), has been the first FDA-approved anticonvulsant drug acting through the activation of potassium channels [30]. Also, flupirtine, an aminopyridine derivative clinically approved as a non-steroidal anti-inflammatory drug (NSAID), was shown to activate neuronal Kv7.2/7.3 channels [31]. Another aminopyridine analogue, named ICA-27243, was shown to selectively activate Kv7.2/7.3 by binding to a site different from the RTG one [32]. ICA-27243 has indeed been thought to bind to a site in the VSD, and its selectivity could be explained on the basis of the greater sequence diversity of the VSD binding site compared to the RTG one among the various Kv7 subunits [32]. Other compounds have been shown to activate the Kv channel by binding to the VSD; among these, we can mention the flavonoid quercetin and the ICA-27243 derivative ztz240, whose complex with Kv7.2 was solved by Cryo-EM (Figure 5), confirming the previously reported evidence that mapped the ICA-27243 and ztz240 binding site to the VSD [11,32,33]. Anyway, most of the drug discovery campaigns aimed at the identification of neuronal Kv7 activators have been looking for ligands binding at the RTG binding site, probably because these latter activators do not influence the activity of cardiac Kv7.1 channels due to the lack of the key tryptophane residue in the binding site (Trp236 In Kv7.2, Trp265 in Kv7.3, Leu266 in Kv7.1) [34,35].

**Figure 6:**
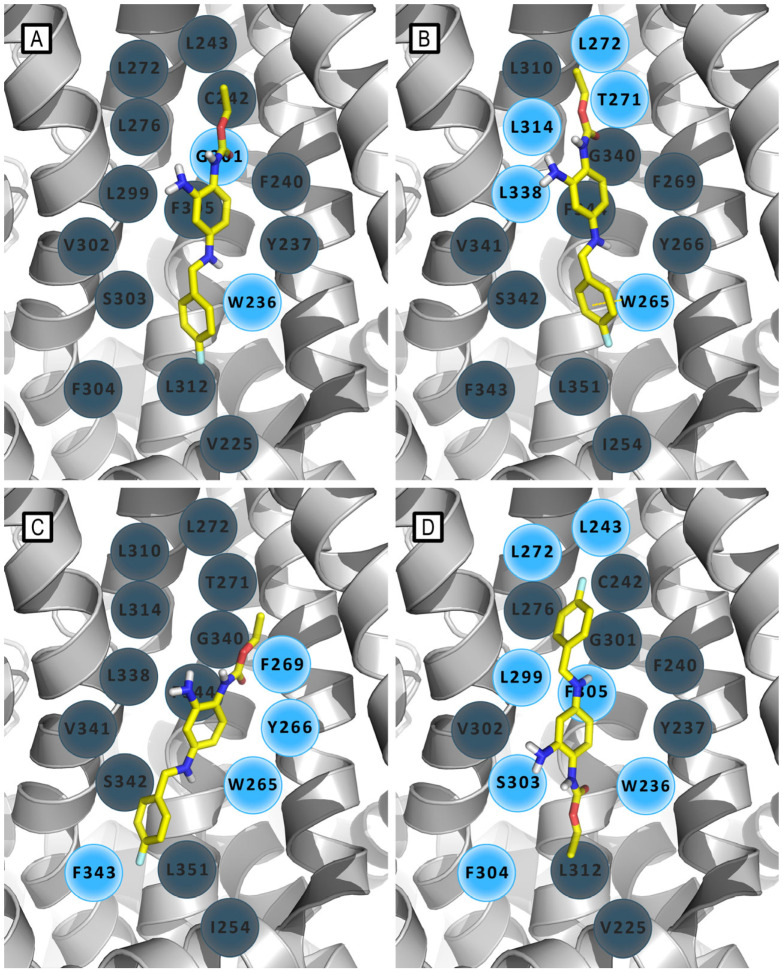
Models of the in silico-predicted interaction patterns of retigabine and its binding orientation within Kv7 channels reported through the years. (**A**) RTG/Kv7.2 model by Wuttke et al. [35]. (**B**) RTG/Kv7.3 model by Lange et al. [36]. (**C**) RTG/Kv7.3 model by Syeda et al. [37]. (**D**) RTG/Kv7.2 model by Kim et al. [38]. In every panel, RTG is depicted by yellow sticks and residues are schematized as gray (not involved in main interactions) and azure (involved in main interactions) circles.

**Figure 7:**
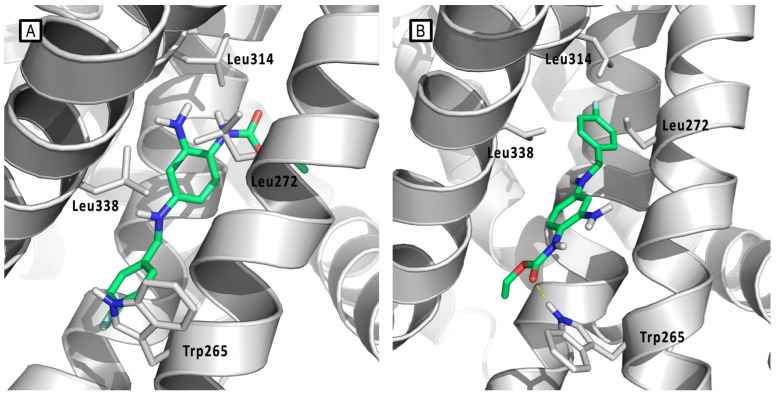
Predicted Kv7.3-bound conformations of RTG according to the models proposed by (**A**) Lange et al. [36] and (**B**) Kim et al. [38]. RTG is represented by green sticks, Kv7.3 channel is represented by gray cartoons, and main residues are reported by gray sticks. H-bonds are represented by yellow dashed lines.

The binding of RTG to Kv7 channels has been studied since 2005, when T. V. Wuttke et al. reported the first molecular modeling study aimed at the identification of the RTG binding site into Kv7.2 channels [35]. They reported a 3D-structural model of the S5 to S6 domain of Kv7.2, generated by SWISS-MODEL, and optimized by SwissPdbViewer (Gromos96), built by homology with the *Methanobacterium thermoautotrophicum* (MthK) channel structure (PDB ID: 1LNQ, open conformation) and to the *Streptomyces lividans* (KcsA) structure (PDB ID: 1BL8, closed conformation). By comparing both channel conformations, the authors revealed the formation of a hydrophobic cleft between S5 and S6 helices, located close to the channel opening, in which the energy-minimized RTG molecule was manually docked. The binding pocket was formed upon the folding of the S6 helix at the gating hinge and a concurrent conformational rearrangement of S5. Contrariwise, in the closed channel, the S5 and S6 helices assumed a parallel orientation that would not allow RTG binding. This model predicted a specific lipophilic interaction between the fluorobenzyl ring of RTG and the aromatic residue Trp236 in the cytoplasmatic region of S5, a prediction matching with the electrophysiological data collected by the authors. Furthermore, the model reported the role of Gly301, located in the S6 helix, which was supposed to act as a gating hinge (Figure 6A and Figure 7A).

In 2009, Lange et al. described a novel homology model of S5-S6 and S1-S6 domains of Kv7.3, modeled by homology with the crystal structures of the corresponding domains of KcsA (PDB ID: 1BL8), MthK (PDB ID: 1LNQ), and of the Shaker-related mammalian voltage-gated potassium channel Kv1.2 (PDB ID: 2A79) [36]. For the realization of this model, a combination of SwissModel and de novo generation of an α-helical peptide chain of the S1–S6 encoding Kv7.3 sequence was used. A Deep View Swiss PDB viewer and BALLView 1.1.1. with Amber and Gromos96 force field were used for the structural optimizations. [39] Docking of energy-optimized RTG conformers to the Kv7.3 homology models was performed by the authors using a geometrical docking approach with the Global Range Molecular Matching program (Gramm v1.03). The analysis of docking results suggested a binding mode for retigabine similar to that previously reported on a Kv7.2 model [35].

This model supports the crucial involvement of residue Trp265 and Leu314 in delimiting the upper and lower margins of the RTG putative binding site and in facilitating ligand anchoring (Figure 6B and Figure 7A). Furthermore, this molecular modeling investigation, supported by experimental analysis, has shed light on further residues affecting RTG binding: the backbone of Thr271 and the side chain of Leu272, both located on the same subunit of the above-mentioned Trp265, and Leu338, which notably extends from S6 of the adjacent subunit, also involved in lining the hydrophobic binding cleft. This pocket, which is located at the interface of two neighbor subunits, was supposed to be present only in the open state of the channel, consistent with the idea that retigabine acts by stabilizing this channel conformation.

In 2015, Syeda et al. reported a slightly different approach to explain how the anti-epileptic drug RTG modulates channel function, acting specifically on the pore modules (PM) of the Kv channels by prolonging the residence time in the open state [37]. The amino acid sequence of PM of the Kv7 channel family and its voltage sensor modules, is similar to that of KvLm, the voltage-gated K^+^ channel from Listeria monocytogenes for which the crystal structure has been solved, limited to the pore region. Therefore, using as a template the open pore conformation of the Kv1.2–2.1 channel (PDB ID: 2R9R) and the closed state of KvLm PM (PDB ID: 4H33) in YASARA (YASARA Bioscience, Wien, Austria), two different structural models of the Kv7.3 pore module were realized. Docking of RTG into the Kv7.3 PM was carried out by using the VINA docking algorithm in YASARA.

As discussed above, this model also describes the Trp265 of Kv7.3, conserved in Kv7.2 (Trp236) to Kv7.5 but absent in Kv7.1, as a mandatory residue for RTG binding. In the closed conformation model, Trp265 is shown to form two distinct intra-subunit π stacking interactions, involving the Phe269 within the same transmembrane helix (S5) and Phe343 located in the S6 helix (Figure 6C). Looking at the open model conformation, the indole ring of Trp265 in a Kv7.3 subunit interacts by two π-π intra-subunit bonds with Tyr266 and Phe269 within the same trans-membrane segment, S5. Therefore, the models suggest that in transitioning from closed to open conformation, a π-π interaction that holds S5 to S6 within the same subunit is lost in the Kv7.3 PM and replaced by an intra-subunit π-π interaction within S5. On this basis, this gating model proposes the π-π interaction between Trp265 in S5 and Phe343 in S6 as a determinant in the stabilization of the closed conformation of the PM. In order to understand how potential RTG interaction with Trp265 could alter the closed state of Kv7.3 PM, Syeda et al. explored the docking conformation of the drug on their opened model structure, finding that in the two lowest-energy docking poses the benzyl ring of RTG interacts with Trp265 by a π-π interaction (Figure 6C) [37]. The bound conformation of RTG within the binding site reported in this work, differs from the earlier model proposed by Lange et al., in which the RTG does not look to form a π-π interaction with Trp265 [36]. Since in all calculation models by Syeda research group, RTG fails to interact with Phe343 in S6, the authors proposed that RTG stabilizes Kv7.3 PM by preventing the formation of a π-π interaction between the Trp265 and Phe343 responsible, according their model, of the closed pore structure stabilization.

In 2015, Kim et al., by the use of unnatural amino-acid mutagenesis found that RTG interaction with Trp takes place by hydrogen bonding [38]. This observation led them to hypothesize a “flip” model for retigabine binding, in which the indole NH of Kv7.3 Trp265 acts as a donor hydrogen bond, that in turn is accepted by the carbamate carbonyl oxygen of RTG (Figure 6D and Figure 7B). This novel RTG/Kv7.3 model was built starting from the RTG/Kv7.3 PD model previously reported by Lange et al., in which RTG was flipped upside down so that its carbonyl oxygen faced the indole NH group of Trp265 thus making the formation of the H-bond possible [36]. The model was then energy-minimized (AMBER03 forcefield) and submitted to MD simulations. It Is worth noting that the “flip” model predicted the correct orientation of Kv7-bound RTG, which was experimentally solved years later (see below).

**Figure 8:**
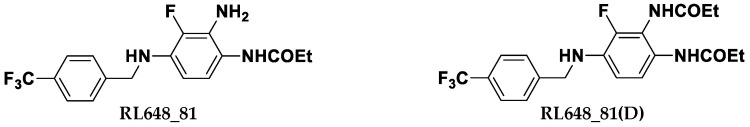
Chemical structures for RL648_81 and RL648_81(D) [39].

Shi et al. reported the investigation of novel RTG analogues, namely, RL648_81 and RL648_81(D) (Figure 8), with different activation potency against Kv7.2 [40]. In this work, the authors first reported a binding mode investigation performed by molecular modeling techniques. In addition, they explored, by MD simulations, the stability and conformational differences of the Kv7.2 upon RTG derivatives binding. 

For the construction of the reported Kv7.2 homology model, the authors used the open state of Kv1.2 (PDB ID: 3LUT). MODELLER was used to generate a preliminary model of the Kv7.2 channel and then the loops were refined by SWISS-MODEL [41]. The molecular docking studies were performed by using Autodock 4.2. For the molecular dynamic simulations, the simulated Kv7.2 models, (i.e., Kv7.2/RTG, Kv7.2/RL648_81, and Kv7.2/RL648_81 (D) were first embedded in an explicit 1-palmi- toyl-2-oleoyl-sn-glycero-3-phosphocholine (POPC) bilayer, which was generated by the CHARMM-GUI server [42]. MD simulations were performed using AMBER16 [11,43]. Analysis of the trajectories bring back the light to the role of the conserved tryptophan residue as element required for the regulation of RTG and its analogues. Additionally, results pointed out the role of other residues constituting the binding pocket (Trp236, Phe240, Leu243, Ala265, Leu268, Leu272, Leu292, Ala295). These data showed that the binding stability of the investigated molecules in the binding site is assured by van der Waals interactions, hydrophobic interactions, H-bond, halogen bond, and π–π stacking.

**Figure 9:**
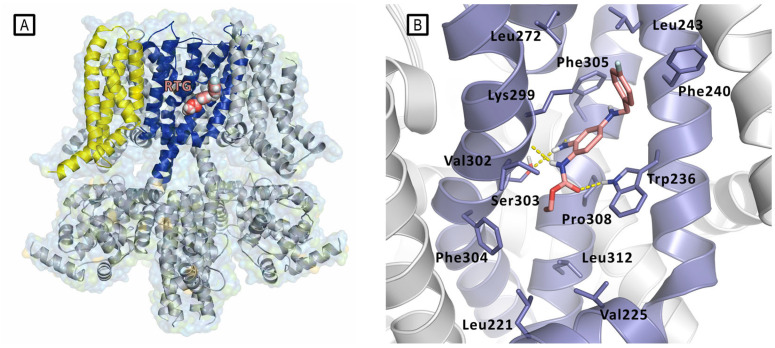
(**A**) Representation of the Cryo-EM structure of Kv7.2 in complex with retigabine (PDB ID: 7CR2). Kv7.2 is represented by cartoons and surface while retigabine is represented by spheres inside the PD binding site. Cartoons are colored as follows: PD of one subunit is colored in blue, while the VSD of the same subunit is colored in yellow. The other subunits are represented in gray. (**B**) Experimental bound conformation of retigabine (pink sticks). Residues of the pocket are represented in light blue sticks, and include Trp236, Ser303, and Lys299 involved in polar interactions (represented as yellow dashed lines) in the pocket comprised between S5 and S6 helices (PD) [11].

Only in 2020, Xiaoxiao et al. reported the first structural studies of the mammalian Kv7.2 channel, providing experimental evidence about the ligand activation mechanisms mediated by small molecules, and thus paving the way for novel antiepileptic and analgesic drug development. Specifically, they reported the first Cryo-EM structure of the Kv7.2 channel in apo-state and in complex with two activators: the above-mentioned N-pyridyl benzamide named ztz240 and the well-known RTG [11]. In the RTG-bound Kv7.2 complex, shaped densities arising from the RTG molecule were observed in a hydrophobic cleft outlined by S5, pore helix, and S6 from the neighboring subunit at the inter-subunit interface in the pore domain (Figure 9). RTG binds to Kv7.2 mainly by the hydrogen bonds network involving the side chains of Trp236, Ser303, and the main chain carbonyl moieties of Leu299 and Phe305. Also, hydrophobic interactions with residues Trp236, Phe240, Leu243, Leu272, Leu299, Phe304, and Phe305 were found to contribute to the complex stabilization. The crystal structure of RTG-bound Kv7.2 also reported an activated voltage-sensing domain and a closed pore. The binding of RTG to the channel mainly triggers conformational change in the pore domain, with a clockwise rotation of upper parts of S5, S6, and the pore helix (viewed from the extracellular side). Indeed, RTG insertion in the interface between S5 and S6 from its neighboring subunit induces a separation of these two helices from each other by almost 1–2 Å. This also causes a rearrangement of the side chain of Trp236 that moves outward to create space for RTG. RTG here is described as an allosteric modulator, binding to the pore domain but modifying the voltage dependence of the channel. An overall superimposable binding mode of RTG was later reported for Kv7.4 [18]. 

To assess the involvement of small molecules in Kv modulation in the different conformational state, Garofalo et al. performed a computational-based characterization of this channel in the two not yet observed functional resting/closed (RC) and activated/open state (AO) [44]. VSD activation was thought to take place stepwise due to the membrane, proceeding from an initial resting VSD configuration in which the pore is closed (resting/closed, RC) to an activated VSD with an open pore (activated/open, AO) [45], passing through a conformational change in the VSD into an activated state with the pore still closed (activated/closed, AC) as later confirmed by the Cryo-EM solved structure of Kv7.2 (PDB ID: 7CR0) [11].

This study provided a useful tool not only to study the structural and dynamic characteristics of the channel, but also the binding of RTG to different PD states. 

The channel was modeled by homology using the Cryo-EM structure of Kv7.1 (PDB ID: 5VMS) as a template and the primary sequence of Kv7.2 from UNIPROT (ID: 043526) [46]. For the MD studies, only the TM region, embedded in explicit phospholipid membranes (palmitoyloleoyl-phosphatidylcholine; ~250 phospholipids per leaflet), was used to perform the simulations. With the exception of the AC state, the models contained also PIP2 inside the membrane to induce conformational changes and K^+^ ions in the channel selectivity filter as seen from X-ray coordinates (PDB ID: 2R9R) [47]. RTG/Kv7.2 starting conformations were obtained by visual inspection of docking poses derived from the clustering method of ensemble docking calculations using Autodock4.2.

The combination of docking and MD simulation of RTG in complex with RC- and AO-Kv7.2 states, suggested that the ligand establishes an extensive interaction network at the same binding site in both analyzed states. Specifically, the binding occurs in a hydrophobic pocket constituted by S5, pore helix, and S6 from the nearby subunit at the inter-subunit interface in the PD. The drug is mainly anchored by hydrogen bonds with the side chain of Trp236, Ser303, and the main chain carbonyls of Leu299 and Phe305. Also, hydrophobic interactions with residues Trp236, Phe240, Leu243, Leu272, Leu299, Phe304, and Phe305 contribute to the stabilization of the complex. 

The many commonalities found between the results of in silico simulations and experimental studies conducted over the years suggest the high potential of computational techniques in this field and provide new insightful tools that might help drug design efforts.

## 4. In Silico-Assisted Design of Kv7.2/7.3 Small Molecule Modulators

Although retigabine has been approved for clinical use in the past, serious side effects associated with long-term treatment conduced to its market discontinuation. Adverse effects are likely due to the poor selectivity profile of RTG among Kv7.2–7.5 channels as well as metabolic oxidation products of its benzenetriamine core. To give an example, the drug activates the isoforms Kv7.4 and Kv7.5, which are not involved in the etiology of hyperexcitability-related neuronal disorders. Since the Kv7.4 channel is mostly expressed in the smooth muscle of the bladder where it regulates contractility, RTG treatment-induced activation of this receptor correlates with reduced contractility causing urinary retention [48]. As a result, there is an urgent need for the identification of potent and selective Kv7.2/7.3 channel activators, which do not affect the functioning of their analogues. 

**Figure 10:**
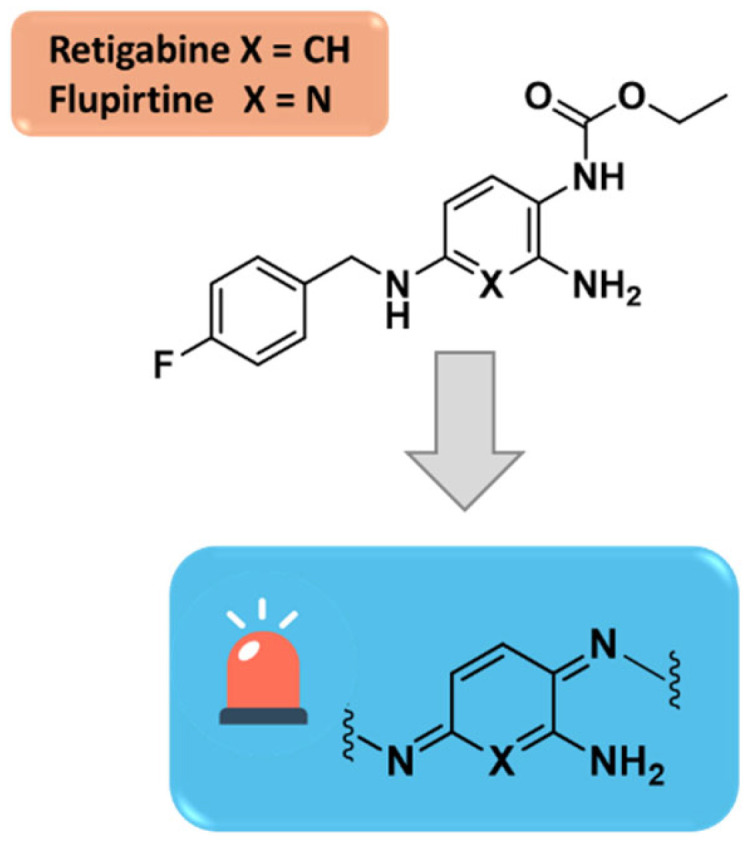
Structure of the reactive metabolite quinone diimines.

In addition, one of the major clinical concerns over RTG is its tendency to induce mucocutaneous and retinal blue-gray discoloration. The molecular mechanism underlying these adverse effects has been questioned for a fairly long time, but in recent years, several research groups have provided evidence supporting a photooxidative RTG degradation mechanism that does not take place in hepatic tissue but is instead the result of a light-induced C-N bond cleavage reaction catalyzed by melanin. Via quinone diimines as intermediates, RTG can dimerize to phenazinium structures, which are the putative causes of blue discoloration of various tissues (Figure 10) [49,50,51,52,53].

Another drawback of the clinical use of RTG is its poor pharmacokinetic: due to the short half-life of the molecule, its lipophilicity (log P = 3.08, which is low if compared to Kv7 agonists whose plasma/brain distribution ratio is higher, such as compound **24**—vide infra—log P = 4.74) and clearance (t1/2 ≈ 6–8 h) that limits plasma concentration and has poor brain penetration, where it becomes necessary for a multiple and high-dose intake per day which negatively affects the patient’s compliance [52].

**Figure 11:**
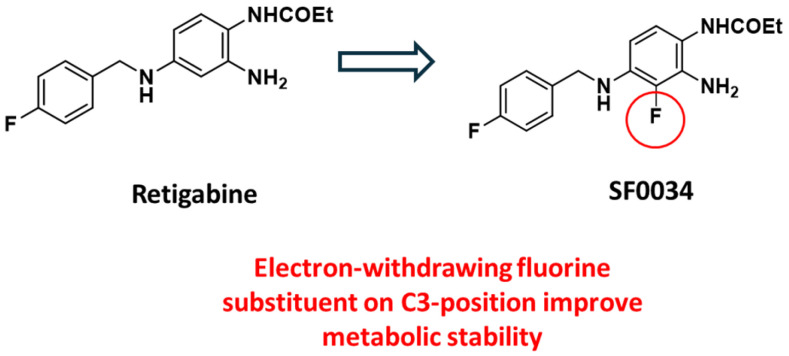
Modification of RTG structure by introduction of fluorine atom on C3-position led to the potent SF0034.

To enhance the RTG pharmacokinetic profile and, at the same time, increase potency toward specific Kv7.2–7.5 channel subtypes, in the last decades, few libraries of retigabine analogues have been designed by pursuing different strategies. Among them, the incorporation of an electron-withdrawing fluorine substituent on the C3-position of the aniline ring has been reported as a first strategy to improve metabolic stability, conducing to the five times more potent SF0034 derivative (Figure 11) [40,54].

Shortly after, the introduction of a CF_3_ group at the 4-position of RTG benzylamine moiety, maintaining the fluorine atom at the 3-position of the aniline ring, led to a new more potent and selective activator of Kv7.2/7.3 named RL648_81 [55]. The binding hypothesis and chemical structure for this compound have been reported above [40]. 

**Figure 12:**
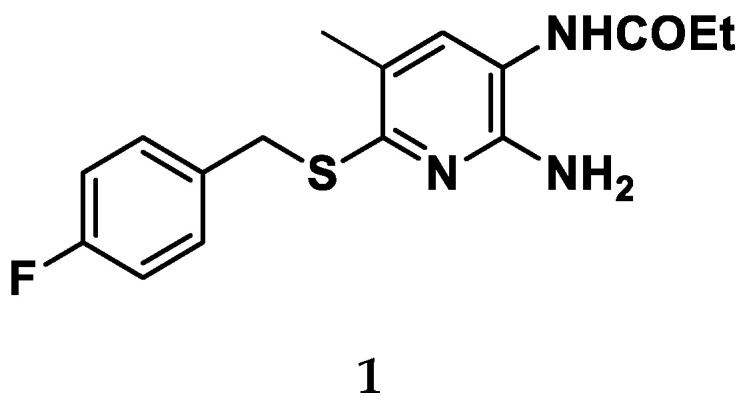
Chemical structure for the sulfide derivative **1**.

A different strategy employed to prevent RTG photodegradation involves substitution of the secondary amine with a sulfur atom (i.e., compound **1**—Figure 12), obtaining sulfide analogues. This simple chemical modification avoids the oxidation process of the aniline and led to the formation of less toxic metabolites disposing the risk of quinone formation [56]. 

**Figure 13:**
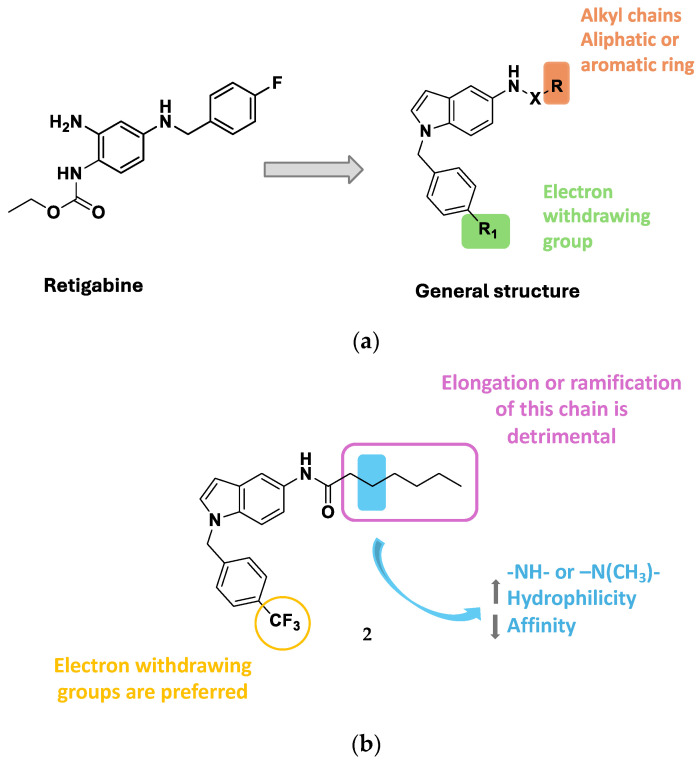
(**a**) General structure for the indole subseries and corresponding SAR maps. (**b**) Chemical structure for the most active compound **2** and relative SAR maps [56].

Ostacolo et al. recently described the design and synthesis of a library of 42 novel conformationally restricted RTG analogues together with the characterization of several of these compounds in terms of chemical stability, selectivity, potency, and in vitro pharmacokinetics [57]. Specifically, new indoline, indole, and tetrahydronaphthalene derivatives were developed by blocking the labile 4-NH_2_ and removing the 2-NH_2_ of the benzene-1,2,4-triamine scaffold (Figure 13). The N1 benzyl group was retained and the effects of substitution with different electron-withdrawal and electron-donating groups were explored. Concerning the linker connecting the amino group at the C5-position (X) with R group, the authors investigated a series of various moieties presenting different electron density distribution, electrostatic potentials, and N-X rotation energy profiles (-CO-, -SO_2_-, -CONH-, -CSNH-, -CNHNH_2_-).

**Figure 14:**
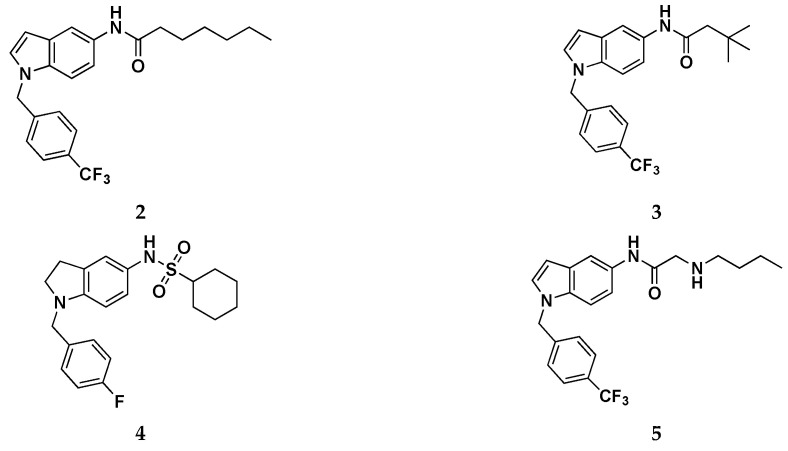
Chemical structures of compounds **2–5**.

**Figure 15:**
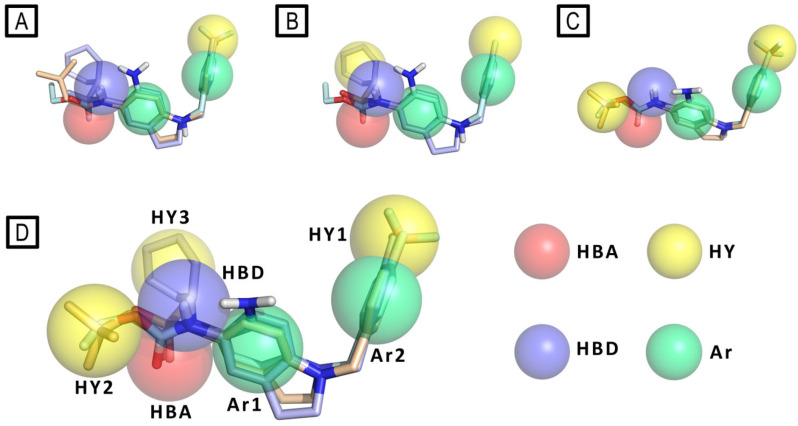
Pharmacophore model (panel (**D**)) derived by merging pharmacophore features of the models (**A**–**C**). RTG is represented as cyan sticks, compound **3** as wheat sticks, and compound **4** as light blue sticks. Pharmacophore features are color-coded as follows: yellow (hydrophobic—HY), green (aromatic—Ar), azure (hydrogen bond donor—HBD), and red (hydrogen bond acceptor—HBA).

Then, pharmacophore modeling was used to investigate the chemical features of the novel active compounds (e.g., **2**, **3**, **4** and **5**—Figure 14). In particular, the final model (D) composed by a hydrogen bond acceptor (HBA), a hydrogen bond donor (HBD), three hydrophobic regions (HY1, HY2, and HY3) and two aromatic regions (Ar1 and Ar2) was obtained by merging three different pharmacophores models: (A) obtained from all active derivatives; (B) from sulfonamide derivatives only; (C) from amide ones only (Figure 15).

The authors stated that the two hydrophobic features, HY2 and HY3, plausibly indicate the presence on Kv channels of a unique large and plastic hydrophobic cleft present in Kv7.2, which could host a variety of substituents as well as more flexible compounds in different conformations, like in the case of the most active sulfonamide derivative **4**. These data result in an agreement with the model of Kv7.2, built by homology with Kv7.1 (PDB ID: 5VMS) [58], presented by the authors, in which the authors highlight the presence of two distinct hydrophobic pockets separated by the residue Trp236 that could host the ligand chemical features HY1 and HY2/3 on the target protein.

Interesting SAR clues have been highlighted for the most promising indole subseries, which are summarized in Figure 13. The role of the R group has been widely explored by introducing alkyl chains of different lengths and aliphatic or aromatic rings. The best results were obtained when a hexyl chain was introduced as an R substituent: the elongation or the ramification of this chain, as well as the ring closure in a cyclohexyl moiety, resulted in being detrimental for Kv7.2 activation, potentially justifiable by an excessive steric hindrance. Furthermore, the replacement of the methylene bridge on the hexyl chains with a substituted or unsubstituted nitrogen atom (compound **5**) led to an improvement of hydrophilicity which did not optimally fit HY2/3, with subsequent activity worsening. Regarding the N-1 benzyl moiety, the best outcome was obtained when an electron-withdrawing group is attached to the para position, as for the most active compounds of the indole series **2** which bears a -CF_3_ moiety. This aspect could be explained by the ability of this tail to fit the AR2-HY1 features of the pharmacophore model. The authors, despite presenting a homology model of Kv7.2, did not run and report in this paper any molecular docking or molecular dynamics simulation of their compounds in complex with their developed Kv.7.2 homology model. Anyway, the most promising compounds from this work, i.e., **2** and **3**, were used as starting points for a follow-up work that made extensive use of target-based in silico studies, using the recently published Cryo-EM structure of the Kv7.2/RTG complex [11,52].

Indeed, in 2022, Musella et al. reported about an in silico guided approach for the design of novel Kv7.2/7.3 activators, followed by the synthesis and characterization for their Kv7 opening ability using both electrophysiology- and fluorescence-based assays [52]. 

The most promising derivatives **2** and **3**, identified in the previous work [57], were submitted to molecular docking and molecular dynamics (MDs) experiments to find out the protein/ligand interactions which are potentially responsible for the enhanced activity (compared to RTG) of these compounds.

**Figure 16:**
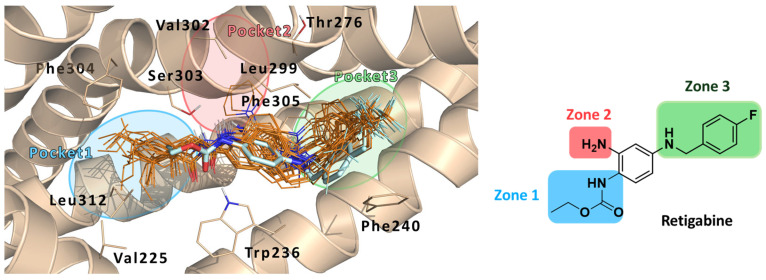
Docking poses of compounds **6–24** (orange sticks) in the PD binding site of Kv7.2 (PDB ID: 7CR2). Experimental Kv7.2-bound conformation of RTG is represented in cyan sticks as reference. The protein main residue is represented, respectively, in wheat cartoon and thin sticks. The three pockets are highlighted as follows: Pocket 1—blue, Pocket 2—red, Pocket 3—green.

This in silico investigation revealed that the hexyl and the 2,2-dimethylbutyl substituents (-R) of **2** and **3**, respectively, fit a hydrophobic region (Pocket 1) lined by the residues Val225, Phe304, and Leu312. These interactions were not found for the parent compound RTG, due to the moderate length of its -R group. MD simulations revealed additional interactions for RTG, 2, and 3 within the Kv7.2 pocket: the -NH_2_ group at the position 2 or RTG forms a hydrogen bond with the polar residue Ser303, which is next to a small hydrophobic pocket (Pocket 2) lined by residues Thr276, Leu299, Val302, Ser303, Phe305, and Ala306. Another pocket (Pocket 3) is instead formed by residues Phe240, Leu243, Leu268, Leu272, Leu275, and Phe305 (Figure 16 – left panel).

**Figure 17:**
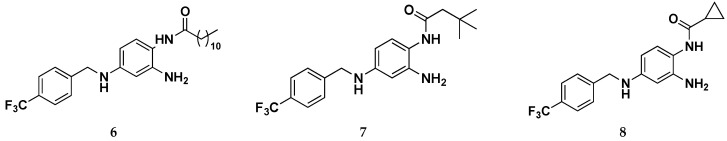

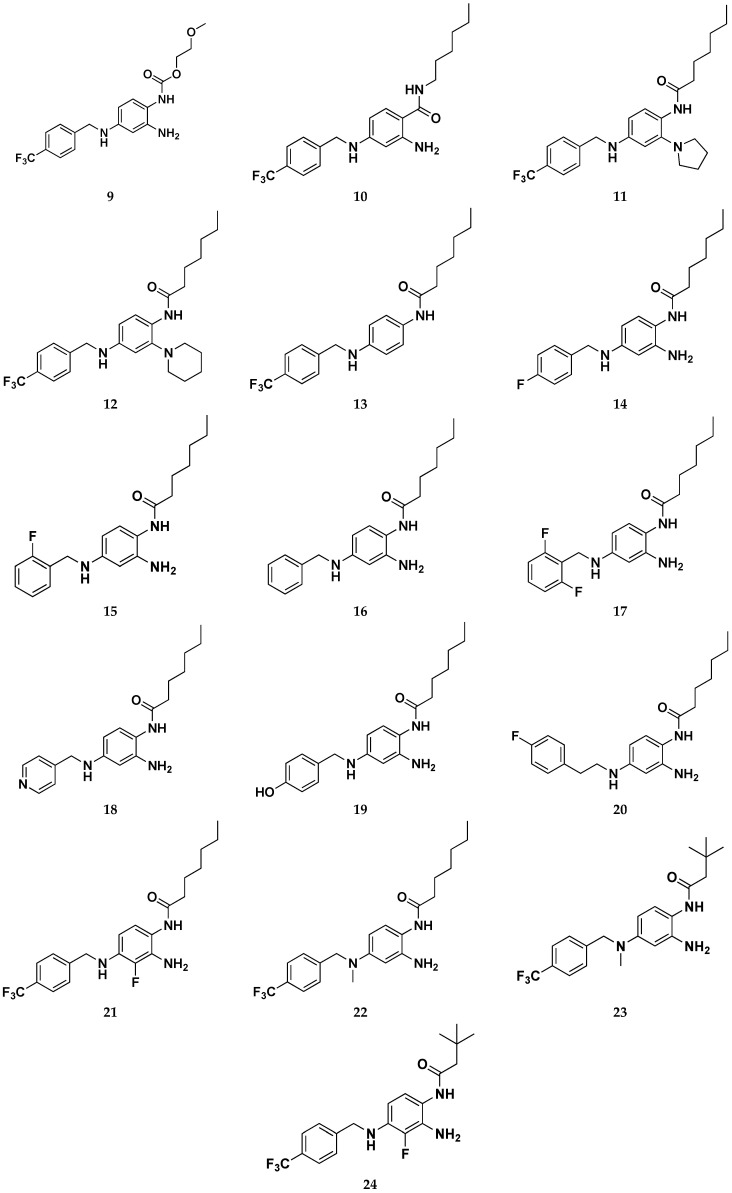
Chemical structures of retigabine derivatives **6–24**.

Focusing on the three binding regions so far identified, the authors developed the first library of RTG analogues by focusing their attention on the three molecular zones (Figure 16—right panel) of retigabine, which relate to Pockets 1–3 on Kv7.2. This investigation made it possible to outline a clear SAR for RTG: zone (1) linear, branched, or cyclic groups (compounds **6**–**8**—Figure 17) are well tolerated when up to six carbon atoms are introduced in R1. The introduction of hydrophilic groups (e.g., ethylene group, compound **9**) and the inversion of the amide bond (compound **10**) are detrimental for activity; zone (2): larger substituents in place of the -NH_2_ group are unable to act as HBDs (e.g., pyrrolidin-1-yl and piperidin-1-yl groups, compounds **11**–**12**) such as substitution with hydrogen atoms (compound **13**), still maintains the activity; zone (3): shifting the fluorine of compound **14**, in position 2 of the benzyl ring (compound 15) or its removal (compound **16**) does not lead to substantial changes in activity. The introduction of 2,6-difluoro benzyl moiety (compound **17**) results in a strong activity. Moreover, substitution of the fluorobenzyl group with pyridine (compound **18**) or hydroxybenzyl groups (compound **19**) led to a complete loss of activity. In a similar way, also increasing the length of the spacer between the fluorobenzyl ring and the secondary amino group at N4 with an extra methylene (compound **20**) led to a complete loss of opening activity.

Continuing these molecular optimization efforts, in the same work, a second library of molecules has been designed to avoid C−N bond photo-oxidative cleavage, which provided isosteric substitution of the -NH- in position 4 with an oxygen atom or methylene groups, molecular modification that failed to activate Kv7.2/7.3 channels. The third group included derivatives replacing hydrogen atoms with fluorine atoms (e.g., compound **21**) at position R2 of the benzene-1,2,4-triamine ring, an approach likely decreasing the reactivity of nitrogen atoms in the C2- and C4-positions. This latter strategy has been successfully used previously to develop the metabolically stable Kv7.2 activators RL-81, which has already been described above [40].

Overall, within this novel series of molecules, three compounds (**22**, **23**, **24**) displayed efficacy as Kv7.2/Kv7.3 channel activators higher than that of retigabine. Intriguingly, compound 24 resulted in being both more photostable than RTG and did not undergo dimerization, as suggested by photostability experiments performed by HPLC analysis. In summary, driven by computational studies and by the SAR information collected, Musella et al. [52] identified a promising lead compound (**24**) which, when compared to retigabine, results in being way more potent as a Kv7 channel activator by in vitro analysis. Furthermore, this compound is characterized by an improved chemical stability and a better pharmacokinetic profile, thus representing an interesting starting point for further molecular optimization.

Recently, Wurm et al. reported a ligand-based approach, which, contrary to the design strategies described above, centered on completely removing structural components responsible for quinoid metabolites formation. For this scope, the substitution pattern of the central aromatic ring of retigabine or flupirtine has been modified in such a way that the two nitrogen atoms are no longer found in the ortho or para position. In summary, the metabolic instable triaminoaryl structure was muted to a nicotinamide scaffold thus paving the way for the development of a novel class of potential Kv7 activators [49,59].

**Figure 18:**
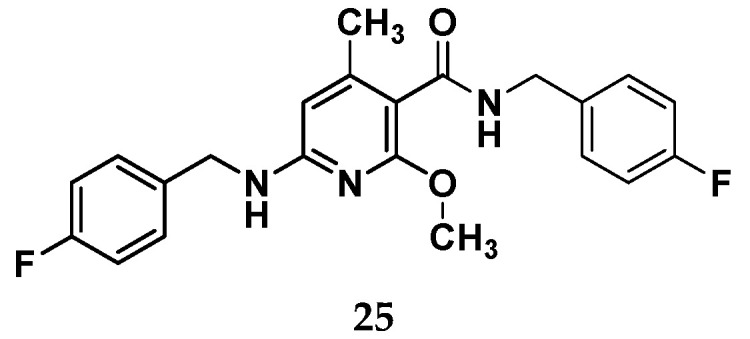
Chemical structure for the nicotinamide derivative **25**.

From the work of Wurm et al., it emerged that when moving to a nicotinamide core, additional structural modifications are requested to maintain a Kv7.2/7.3 opening activity, e.g., the introduction of an additional benzylic amide side chain together with a methyl group on a pyridine core as in compound **25** (Figure 18) [49].

**Figure 19:**
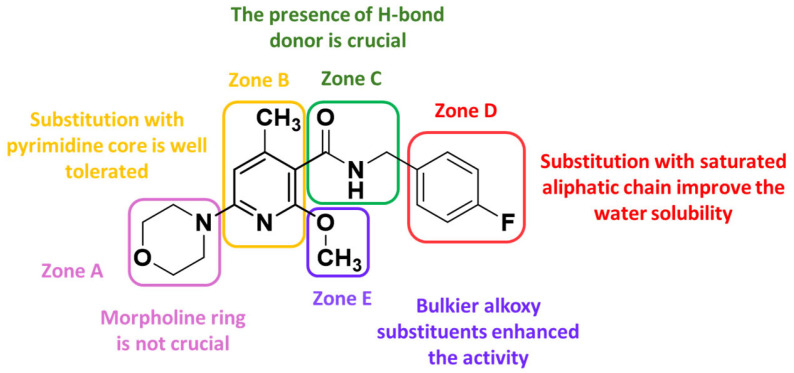
Chemical structure of the lead compound **26** and relative SAR maps [58].

Although this compound showed a good potency profile, it presents the drawback of a high lipophilic character and a low concentration of sp^3^-hybridized carbon atoms that correlate with poor water solubility. This aspect has hindered the toxicity tests, suggesting that further structural optimizations for this species is needed. To avoid this shortcoming of compound **25** and by recurring to a molecular hybridization approach, a novel Kv7.2/7.3 activator candidate (**26**) has been developed and is then used as a starting point for a new design. Indeed, the chemical structure of **26** was split into five distinct zones, each of which was stepwisely replaced with different structural motifs to gain more SAR information (Figure 19).

**Figure 20:**
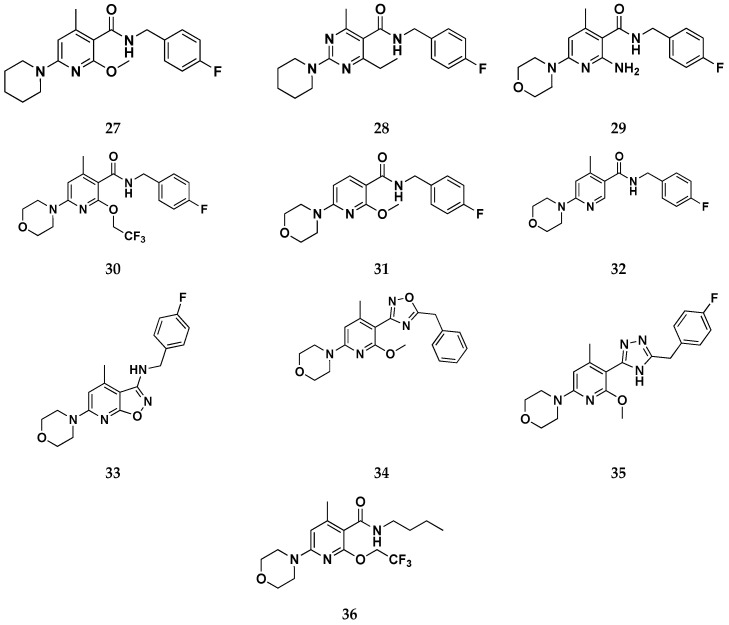
2D-chemical structures for the novel Kv activators **27–36** [58].

First, derivatives of compound **26** with modifications in zone A were designed and synthesized with the aim to elucidate the role of the morpholine ring and its tertiary amine in the binding within the Kv7.2/7.3 binding site. It was found from in vitro experiments that both heteroatoms of the morpholine ring are not crucial for Kv7.2/7.3 binding, since the substitution of oxygen with a methylene group (compound **27**—Figure 20) resulted in an enhancement of activity. These data are in good agreement with molecular docking results that place the morpholine ring in a hydrophobic cleft with no involvement of the oxygen atom in any H-bonding interaction. 

Second, the importance of the pyridine core (zone B) was investigated by substitution with a pyrimidine ring (compound **28**), leading to a potent nanomolar Kv7.2/7.3 activator. Also in this case, there is a matching with docking studies results, which predicted a very similar binding mode for both pyridine and pyrimidine derivatives, although no further selective interaction mediated by the additional pyrimidine nitrogen atom was detected. 

**Figure 21:**
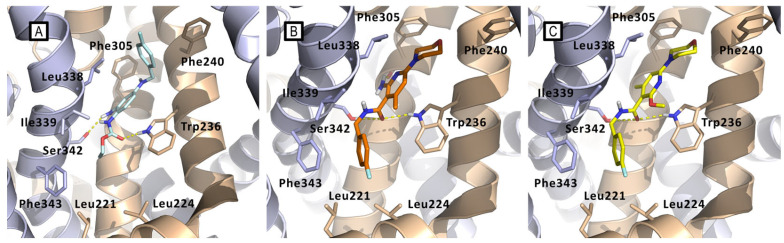
Comparison between the docking-predicted Kv7.2/7.3-bound conformations of (**A**) RTG (cyan sticks); (**B**) compound **29** (orange sticks); (**C**) compound **26** (yellow sticks). Kv7.2/7.3 subunits are represented as wheat and azure cartoons, respectively. Main residues of the pocket are represented as sticks. All three compounds maintain the H-bond interaction with Trp236, showing only a slight displacement of the central pyridine ring in respect to the core phenyl ring of RTG. Ser342 makes H-bonds with the three compounds, but **26** and **29** make it by their carbonylic oxygen rather than with the amino group as in the case of RTG. The amino group of **29** is instead directed toward a hydrophobic spot, where even the methyl substituent of **26** is found. The 4-fluorobenzyl group of **26** and **29** lay in the large hydrophobic pocket formed by Leu221, Leu225, and Phe343.

Regarding the exploration of zone E, identifiable in the C2-position of the pyridine core, different approaches were followed. At first, with the final aim to enhance solubility in a water medium, the primary amino group shared by flupirtine and retigabine has been reintroduced resulting in compound **29**. It thus emerged that the primary amino function is clearly disadvantageous in nicotinamide derivatives, although its presence is beneficial for retigabine and flupirtine. Modeling studies explained this result, showing that the amino group of **29** affects the binding mode within the pocket if compared to retigabine and the parent compound **26** (Figure 21). In addition, the primary amino group of **29** (Figure 21B) is not involved in the H-bonding interaction with Ser341 as seen for retigabine, due to a slight shift of the central pyridine core.

Later, a different strategy was carried out on attempting to improve the Kv7.2/7.3 opening ability by modifying the alkoxy group of **26**. Further derivatives embedded with bulkier alkoxy substituents were synthesized, leading to an excellent improvement of activity value. Indeed, the opening activity of lead compound **26** was successfully enhanced by the introduction of a 2,2,2-trifluoroethoxy group as in compound **30**, which was shown to be as much as 150 times more potent than flupirtine and **20** times more potent than retigabine.

Unlike the role of the pyrimidine core, a solid conclusion can be made concerning the role of the methyl group in zone B: the influence of the methyl group on the opening activity is strictly influenced by the presence of the second ortho substituent of the amide group. Indeed, the analogous compound to the parent **26**, differing uniquely for the absence of the methyl group (compound **31**), resulted inactive in the in vitro testing up to a concentration of 20 μM. The authors speculated that the presence of substituents in both ortho positions, with respect to the amide group, is necessary to induce a favorable molecular geometry to the binding process, in which the amide group is rotated out of the aromatic plane. Indeed, it seems that the presence of a single methoxy substituent (compound **31**) induces an unprivileged coplanar conformation of the amide group through the establishment of an intramolecular H-bond interaction. This effect could not be verified with a single ortho substituent methyl group (compound **32**) justifying the reason why the methylated analog retains at least a minimum activity. 

Concerning the role of the N-substituted amide group, it was supposed that in the bound state conformation it could be rotated out in respect to the pyridine plane. Furthermore, the binding mode hypothesis suggested that this group could be involved in direct interactions with the KV7.2/3 binding site as both an H-bond acceptor and donor. This could explain why the incorporation of zone C into conformationally restricted rings, such as isoxazolo [5,4-b]pyridine system (compound **33**) or oxadiazole (compound **34**), conduced to inactive compounds. Furthermore, both of these latter ring systems are not able to act as a hydrogen bond donor, which gives an elucidation about the primary role of the amide bond in the binding process. Further support to this assumption is given by the cyclic amide bioisosteres, compound **35**, which showed superior efficacy if compared with **33** and **34**. Specifically, docking simulations suggested that the triazole ring goes to perfectly mimic the amide bond, establishing both H-bond interactions usually mediated by this group, and an additional one that involved the polar residue Ser342.

The amide side chain in zone D, coinciding with a benzyl function in lead compounds **25** and **26**, was found to be responsible for the poor water solubility of the present derivatives. However, since it is known that incorporation of polar functions in this area is not a useful strategy, the authors attempted substitution with saturated aliphatic chains in order to increase the fraction of sp^3^ carbons and thus the water solubility. The best outcomes have been obtained by introducing an n-butyl side chain, conducing to the best compound of this study, **36**, both in terms of efficacy and physicochemical properties.

In 2023, Zhang et al. carried out a structure-based virtual screening of the Specs and ChemDiv databases in search of Kv7.2 activators, using the Cryo-EM structure of Kv7.2 (PDB ID: 7CR2) [11] as a docking target [60]. Prior to the docking simulations, which were run using Schrödinger Glide, the database compounds were filtered to remove pan-assay interference compounds and those violating the Lipinski rule of five. The selected molecules were then submitted to Schrödinger Epik to predict their protonation states at pH 7.0 ± 2.0. The virtual screening was performed in a stepwise fashion using Glide high-throughput and standard and extra precision mode, in a funnel that finally led to the selection of 15 compounds for the electophysiological assays, that in turn identified three compounds able to enhance the Kv7.2 current amplitudes and produce a hyperpolarization shift of the voltage-dependent activation curve. The most potent compound among the three, i.e., Ebio1, displayed a peculiar electrophysiological profile, different from RTG, that led the authors to hypothesize a novel mechanism of action. Indeed, whole-cell electrophysiology and patch-clamp assays revealed that Ebio1 acts by enhancing both the probability of channel opening (PO) and the conductance of the Kv7.2, differing from the well-known activators.

**Figure 22:**
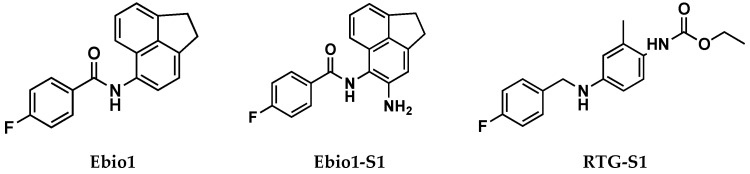
Chemical structures for Ebio1, Ebio1-S1, and RTG-S1.

The authors thoroughly investigated the idea of the alternative mode of action through an extensive use of Cryo-EM, MD simulations, and through the design, synthesis, and electophysiological characterization of two RTG and Ebio1 derivatives, i.e., Ebio1-S1 and RTG-S1 (Figure 22). Ebio-S1 differs from its parent compound Ebio1 for the presence of a -NH_2_ substituent on the dihydroacenaphthylene group, while RTG-S1 differs from its parent compound RTG for the substitution of the -NH_2_ substituent at the core phenyl group with a methyl group. Cryo-EM structures of apo (PDB ID: 7CR0), RTG-bound (PDB ID: 7CR2) [11], Ebio1-bound (PDB ID:8IJK), and Ebio-S1-bound (PDB ID: 8X43) Kv7.2, clearly suggested that Ebio1-mediated channel activation is operated through a twist-to-open-mechanism in which Ser303 and Phe305 move away from each other and S314 and L318 side chains move apart from the ion pathway, with the S6 helices moving away from the pore central axis with a twisting motion [60].

**Figure 23:**
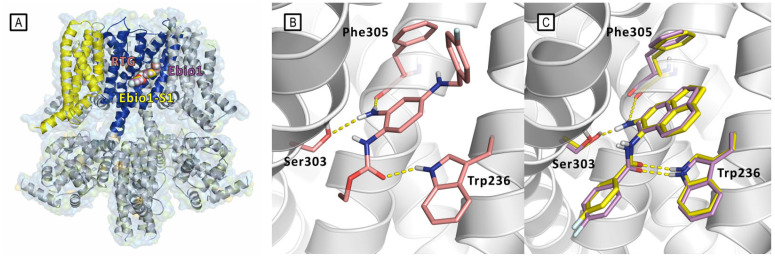
Bound conformations of RTG (PDB ID: 7CR2), Ebio1 (PDB ID: 8IJK), and Ebio1-S1 (PDB ID: 8X43) [11,60]. (**A**) Ligand-binding poses represented by spheres inside the PD binding site. Kv7.2 is represented by cartoons and surface; cartoons are colored as follows: PD of the ligand-bound subunit is colored in blue, while the VSD of the same subunit is colored in yellow. The other subunits are represented in gray. (**B**) Details of the RTG/Kv7.2 interaction. RTG, Trp236, Ser303, and Phe305 are represented as pink sticks. H-bonds are represented by yellow dashed lines. (**C**) Details of Ebio1/Kv7.2 (yellow sticks) and Ebio1-S1/Kv7.2 (magenta sticks) interactions. H-bonds are represented by yellow dashed lines.

Even though Ebio1 fits cozily in the same cleft of RTG, composed by S5, S6 helices, and an S4–S5 spacer (Figure 23), the different chemical structures of these two activators may arouse the twist-to-open activation mechanism triggered upon Ebio1 binding. Looking at the Cryo-EM structure of RTG-bound Kv7.2 (Figure 23B), the free primary amino group of the pyridine core faces toward the S6 segment, forming an H-bond, with the side chain of Ser303 of one S6 helix and the oxygen atom from the Phe305 backbone of the neighbor S6 segment. On the contrary, Ebio1 cannot form H-bonds with either Ser303 or Phe305 (Figure 23C), thus leaving S6 more dynamic, and the channel activation gate can become wider. RTG-S1 and Ebio-S1 were used as tools to further confirm the role of ligand H-bonding to Ser303 and Phe305 in the twist-to-open mechanism. In particular, docking and molecular dynamics simulation suggested, as confirmed by electophysiology studies, that RTG-S1, which is not capable of making any hydrogen bond with Ser303 or Phe305, has a behavior that is more similar to Ebio1 than to RTG, being capable of triggering the twist-to-open. On the other hand, molecular docking and molecular dynamics simulations, Cryo-EM, and electophysiology studies showed that Ebio-S1, which is capable of H-bonding to Ser303 and Phe305 (Figure 23C), behaves similarly to RTG.

## 5. Conclusions

A lot of solid scientific evidence suggests the use of Kv7.2/7.3 modulators as a new pharmacological strategy to tackle several neurological diseases that are usually refractory to the current medical treatments, particularly intractable epilepsies during childhood. Moreover, the use of Kv7.2/7.3 modulators is claimed to be one of the most valuable approaches to develop effective tools for precision medicine. In this context, for a long period, the absence of a resolved crystal structure of the Kv7.2/7.3 channels’ binding site has represented the major issue. The use of in silico methods has partially mitigated this setback, providing several clues that have been profitably used. What appears clear from the literature review is that the most important developments in the field have been produced when a multidisciplinary approach has been used, combining computational chemistry, electrophysiology, medicinal chemistry, and pharmacology. Nowadays, the results that can be obtained by these multidisciplinary research lines are strongly improved by the outbreak of Cryo-EM techniques, which provide a detailed mapping of the Kv7.2/7.3 binding site, together with solid clues about these channels and their activation mechanisms. This is why, in the very next few years, we will surely see further development in this field, with the identification of more and more drug candidates accessing the clinical stages.

## Data Availability

Not applicable.
